# Diurnal variation in fecal concentrations of acid-detergent insoluble ash and alkaline-peroxide lignin from cattle fed bermudagrass hays of varying nutrient content

**DOI:** 10.1186/s40104-015-0024-1

**Published:** 2015-06-02

**Authors:** Juvenal Kanani, Dirk Philipp, Kenneth P Coffey, Elizabeth B Kegley, Charles P West, Shane Gadberry, John Jennings, Ashley N Young, Robert T Rhein

**Affiliations:** Division of Agriculture, University of Arkansas, Fayetteville, AR 72701 USA; Department of Plant and Soil Science, Texas Tech University, Lubbock, TX 79409 USA

**Keywords:** Digestibility, Fecal sampling time, Internal markers, Bermudagrass, Cattle

## Abstract

**Background:**

The effect of time of fecal sampling on the accuracy of acid-detergent insoluble ash (ADIA) and alkaline-peroxide lignin (APL) for the prediction of fecal output (FO) in cattle was evaluated. Eight ruminally cannulated cows (594 ± 35.5 kg) were allocated randomly to 4 bermudagrass [*Cynodon dactylon* (L.) Pers.] hay diets markedly different in crude protein concentration (79–164 g/kg) with 2 replicates per diet for 3 periods. Cows were offered hay individually at 20 g DM/kg of body weight daily in equal feedings at 08:00 and 16:00 h for a 10-d adaptation period followed by 5-d of total fecal collection. Fecal grab samples also were taken each day during the fecal collection period at 06:00, 12:00, 18:00, and 24:00 h either directly from the rectum or from freshly voided feces. Samples were composited within cow and time across the 5 d total fecal collection period. Additionally, forage, ort, and fecal samples were analyzed for concentrations of APL and ADIA.

**Results:**

Fecal concentrations of ADIA and APL were not affected by sampling time (*P ≥* 0.22), even though diet affected (*P* < 0.01) fecal ADIA and APL concentrations. There were no diet × sampling time interactions (*P* ≥ 0.60). Estimates of FO and dry matter digestibility (DMD) from ADIA and APL were not affected (*P* ≥ 0.16) by sampling time or the diet × sampling time interaction (*P* ≥ 0.74). Estimates of FO and DMD from markers from different sampling times or all different combinations of sampling time were not different (*P* ≥ 0.72) from those of total collection among internal markers.

**Conclusion:**

Little variation in concentrations of ADIA and APL in daily fecal excretion across time increases flexibility in fecal grab sampling schedules for predicting FO and DMD.

## Background

Due to the expense and difficulty involved in collecting total feces across numerous forages for *in vivo* measurement of dry matter intake (DMI), fecal output (FO) and DM digestibility (DMD) in ruminant animals, external and internal markers can be employed to estimate feed output [1–3]. Internal markers, constituents of feed that are neither digested nor absorbed by the animal [2], are a preferable option for estimating DMI, FO, and DMD because they are expected to flow through the gastrointestinal tract with the digesta they mark [4, 5].

Several studies have detected diurnal variation in the fecal concentration of external markers [6], but few studies [5, 7] have even evaluated diurnal fecal concentration patterns of internal markers. Bias in estimating fecal excretion can have two sources. First is failure of markers to be totally recovered or assayed equally in diet and feces samples (long term bias), and second, failures or inconsistencies in obtaining representative samples of the feed or total feces excreted [5, 8]. Diurnal fecal variation can be overcome by collecting multiple samples throughout the day to provide a composite sample in which the marker concentration is close to the concentration of the entire day [6] provided samples are composited based on marker concentration, not on a DM or wet matter basis. To alleviate the tedious work of total collection for estimating apparent DMD of cattle feeds, information is needed on the diurnal variation of internal markers during a 24-h period to determine whether or not sampling time affects the ratio of marker to the component of interest.

In a recent study [9], alkaline peroxide lignin (APL) and acid-detergent insoluble ash (ADIA) were found to be suitable internal markers to predict FO and DMD by cattle fed bermudagrass hays across a range of CP concentrations when based on total fecal collection of feces, not on fecal grab samples. The objective of this study was to evaluate the effect of time of sampling of feces on the accuracy of ADIA and APL in predicting FO and DMD by cattle fed bermudagrass hays that differed in CP concentration.

## Materials and methods

The site of the study, the experimental layout, and diet treatments were described previously [9]. Eight ruminally cannulated cows (594 ± 35.5 kg) were allocated randomly to 4 bermudagrass hay diets categorized by protein concentration as being low (L), medium low (ML), medium high (MH), or high (H) (i.e.,79, 110, 130, and 164 g CP/kg DM, respectively). Diets were offered in 3 periods to provide 2 replicates per diet per period that resulted in 24 total *in vivo* observations. Diets were rotated across cows between periods. Cows were offered hay individually at a rate of 20 g DM/kg of body weight daily in equal feedings at 08:00 and 16:00 h for a 10-d adaptation followed by 5-d for total fecal collection in each period. Hay, orts, and feces from each period were analyzed for APL, and ADIA concentrations. Actual DMI, DMD, and FO were determined based on the amount of hay offered and orts, and of feces excreted. Recovery of APL, and ADIA were expressed as the ratio of the quantity of marker excreted per unit of marker consumed. Values of DMI, DMD, and FO based on total fecal collection also were described in detail previously [9]. All procedures were approved by the Institutional Animal Care and Use Committee of the University of Arkansas (IACUC approved protocol #10016).

### Fecal grab sample collection and preparation

Fecal grab samples (approximately 300 g wet matter for each sample) were taken 4 times daily (denoted in subscripts as 1 = 06:00, 2 = 12:00, 3 = 18:00, and 4 = 24:00 h) directly from the rectum of each cow or from freshly excreted feces. Samples were oven-dried at 50 °C immediately. Feces from the grab samples, composited by cow and time of sampling within period, were ground through a 1-mm screen of a Wiley mill (Arthur H. Thomas Scientific, Philadelphia, PA, USA).

### Chemical analysis of APL, and ADIA in fecal grab samples

Ground hay, ort, and fecal grab samples were analyzed for ADIA [10] using the ANKOM procedure (ANKOM Technology Corp.#F57, Fairport, NY, USA), by first analyzing a 0.5 ± 0.01 g sample for ADF. The bags had a pore size of 25 ± 10 μm. The ADF residue was ashed in a muffle furnace (Thermolyne Sybron, Thermolyne Corporation, Dubuque, IA, USA) at 500 °C for 8 h. Alkaline-peroxide lignin analysis used a modified procedure [11, 12], for which each 0.5 ± 0.01 g sample was placed in a filter bag (ANKOM Corp., #F57) instead of using filter tubes and filter paper, and incubated in an alkaline-hydrogen peroxide (pH = 11.5) solution for 24 h, and rinsed to neutral pH with hot distilled water after incubation. The alkaline-hydrogen peroxide residue was analyzed sequentially for ADF and ADL to obtain APL concentrations in fecal grab samples.

### Calculation of DMD and FO using ADIA, and APL from fecal grab samples

The concentrations of APL and ADIA in consumed forage and feces, measured fecal output, and apparent DMD were reported in detail in the previous article [9]. The estimated DMD using the fecal grab samples taken at different times (1, 2, 3, and 4) was calculated by the following formula:

1 $$ DMD\ \left(g/ kg\right) = 1000\ \left(g/ kg\right) \times \left[1\ \hbox{--}\ \left({M}_{fd}\left(g/ kg\right)\ /\ {M}_{ftime}\left(g/ kg\right)\right)\right] $$

where M_fd_ is the marker concentration in consumed feed; M_ftime_ is the marker concentration in each fecal grab sample at a particular sampling time.

Estimates of FO of DM by fecal grab samples taken at different times were calculated according to the following expression:

2 $$ FO\left(g/d\right) = DMI\ \left(g/d\right) \times \left({M}_{fd}\left(g/ kg\right)\ /\ {M}_{ftime}\left(g/ kg\right)\right) $$

### Statistical analysis

Data for marker concentrations in grab samples, and FO and DMD estimates derived from the marker concentration at different sampling times and their different combinations (15) were analyzed as a 4× 3 Youden Square design [13] using PROC MIXED of SAS (SAS Inst. Inc., Cary, NC, USA, 2009). Based on 4 sampling times, the resulting single sample times and all possible 2-, 3-, and 4-way combinations of these 4 sampling times resulted in 15 different combinations of sampling time means to be compared to the in vivo total collection data. These values were compared to determine the variation in marker concentrations at various times as well as to determine how close the concentrations of markers in the grab samples were to those obtained by subsamples of total feces, and to determine which time or combination of times of sampling would provide estimates of FO and DMD closest to those of total fecal collection. Effects of diet, marker, sampling time, and the 2- and 3-way interactions among diet, marker, and sampling time were included in the model and significance was noted at *P* < 0.05. In cases where no marker × time or diet × marker × time interaction was detected, each individual marker was analyzed separately to detect potential diet × time interactions within each individual marker. The model included diet, time, and the diet × time interaction.

## Results and discussions

The analysis of the entire data set (period = 3; diet = 4, cow within diet within period = 2, time with all sampling time combinations = 15, marker = 2; n = 720) where diet, marker and time all were included in the model revealed that diet (n = 4), marker (n = 2) and the diet × marker interaction (n = 8) affected (*P* < 0.001) estimates of FO and DMD, but time of sampling had no effect (*P* ≥ 0.96) on the prediction of FO and DMD. In addition, the interactions of marker × time, diet × time, and diet × marker × time of sampling were not significant (data not shown; *P* ≥ 0.99). Therefore, it was concluded that the two markers behaved similarly regarding their prediction of FO and DMD. Thus, data for each individual marker for which diet, time, and diet × time interaction were included in analysis of the model as discussed below.

### Marker concentration in feces by sampling time

The chemical composition of the diet treatments and values of DMI, DMD, and FO derived from total fecal collection were presented and discussed in our recent article [9]. Concentrations of internal markers in feces and effects of time of grab-sampling are presented in Table 1. No diet × time of sampling interaction (*P* ≥ 0.60) was detected for either marker. The concentrations of ADIA and APL were not affected by sampling time (*P* = 0.45 and *P* = 0.22, respectively), even though diet affected (*P* < 0.01) fecal ADIA and APL concentrations.

**Table 1:** Mean fecal concentrations (g/kg dry matter, DM), and estimates of fecal output (FO, g/d), and dry matter digestibility (DMD, g/kg DM) using acid-detergent insoluble ash (ADIA), and alkaline-peroxide lignin (APL) from feces sampled at different times compared with actual fecal concentrations, FO, and DMD values from total collection (TC)

Marker	Time of sampling^a^						*P*-value^c^		
	1	2	3	4	TC	SEM^b^	D^d^	T	D × T
Fecal concentration (g/kg DM)									
ADIA	59	58	61	58	58	4.0	<0.01	0.45	0.60
APL	55	59	58	58	56	3.5	<0.01	0.22	0.92
FO (g/d)									
ADIA	4036	4069	3928	4073	4207	297.9	<0.01	0.64	0.78
APL	4105	3903	3907	3922	4207	285.9	<0.01	0.38	0.99
DMD (g/kg DM)									
ADIA	557	554	574	551	539	22.9	<0.01	0.16	0.86
APL	550	576	571	574	539	17.8	0.30	0.21	0.98

^a^Different sampling times (1 = 06:00, 2 = 12:00, 3 = 18:00, and 4 = 24:00 h)

^b^SEM, standard error of the mean

^c^D, diet; T, sampling time; D × T, diet by sampling time interaction

^d^D, diet consisted of low (L) CP hay (CP = 79 g/kg DM); medium low (ML) CP hay (CP = 111 g/kg DM); medium high (MH) CP hay (CP = 131 g/kg DM); and high (H) CP hay (CP = 164 g/kg DM)

Sampling time has had no effect on APL concentrations in feces in a previous study [7]. Fecal lignin concentrations were relatively uniform within day and were not impacted over a sampling schedule of 3-h intervals for 48 h [14], and daily variation in lignin (72 % sulfuric acid) content of feces from sheep on a diet of timothy [*Phleum pratense* L.] hay was also very small [15]. Furthermore, no interaction between diet and time was detected in the latter study. No significant diurnal or day-to-day variation was detected for acid-insoluble ash [16, 17] and ADIA [18] concentrations in feces in other studies. Concentrations of indigestible ADF and indigestible NDF were similar among samples taken 4 times daily (13:00, 07:30, 13:30, 19:30 h) when compared with indigestible ADF and indigestible NDF concentrations in total fecal collection [8]. Fecal excretion patterns were uniform for indigestible DM, indigestible NDF, and indigestible ADF in a digestion trial with cattle fed diets including elephant grass (*Pennisetum purpureum* Schumach.) silage, corn (*Zea mays* L.) silage, and signal grass (*Brachiaria decumbens* Stapf) hay [5]. Moreover, fecal indigestible ADF content from grazing sheep varied little within these periods across 5 d [19].

The oscillation rate, which is calculated as the difference between the maximum fecal concentration of a marker and the minimum divided by the overall mean fecal marker concentration [5], provides information on the variability of the marker around the mean fecal concentration. In this study, the oscillation rate was 5.1 % for ADIA and 7.0 % for APL. Similar oscillation rates (6.6, 5.8, and 8.5 %) have been reported for other internal markers such as indigestible DM, indigestible NDF, and indigestible ADF [5]. Ideal markers should flow similarly to and be physically associated with the digesta they mark [4]. Internal markers, that are natural components of feeds are expected to flow with the digesta through the gastrointestinal tract of the animal [5, 8]. This explains why variation in fecal content of the internal markers studied across different time was minimal as the marker is a dietary component. Variations in marker concentrations in feces could result from differences in diet and digestibility, and the feeding frequencies [20]. In addition, the natural event of transit and degradation of ingested feed, although continuous in the rumen, may not be constant throughout the remainder of the digestive tract [5, 8].

### Fecal output estimation and digestibility by sampling time

**Fig. 1:**
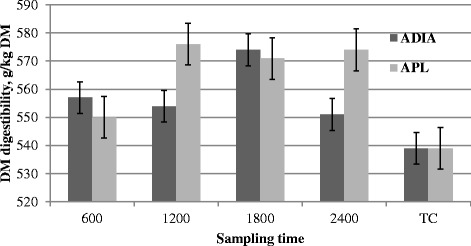
Comparison of DM digestibility values from fecal sampling time and those from total fecal collection

Estimates of FO and DMD at different fecal grab sampling times (1, 2, 3, and 4) are presented in Table 1. Diet affected (*P* < 0.01) the predictions of FO using ADIA and APL, but time of sampling and diet × time were not significant (*P ≥* 0.38 and *P* ≥ 0.78, respectively) for the prediction of FO by ADIA and APL. Diet affected (*P* < 0.01) the prediction of DMD based on ADIA, but not (*P* = 0.30) when based on APL. Time of sampling (*P ≥* 0.16; Fig. 1) and diet × time (*P* ≥ 0.86) had no effect on the prediction of DMD by either ADIA and APL.

Estimates of FO derived using the mean marker concentrations across the 4 fecal grab samplings per day for the 2 internal markers (mean of the four grab sampling times for ADIA (ADIA_1234_) and APL (APL_1234_) and the FO value obtained by total fecal collection procedure were not different from each other (*P* = 0.20, Table 2). Although diet affected (*P* < 0.01) estimates of FO, the diet × marker interaction did not affect estimates of fecal output (*P* ≥ 0.90).

**Table 2:** Comparison of in vivo dry matter digestibility (DMD, g/kg DM) and fecal output (FO, g/d) with estimates obtained by two internal markers using the mean of 4 fecal grab samples per day

	Marker^a^				*P*-value^e^		
Item^b^	ADIA_1234_	APL_1234_	TC^c^	SEM^d^	D^f^	M	D × M
FO (g/d)	3986	3934	4207	113.0	<0.01	0.20	0.90
DMD (g/kg)	561	571	539	15.8	<0.01	0.11	0.74

^a^ADIA, acid-detergent insoluble ash; APL, alkaline-peroxide lignin. Each value represents the mean from four grab samples per day (06:00, 12:00, 18:00, and 24:00 h)

^b^FO, fecal output; DMD, dry matter digestibility

^c^TC, total fecal collection

^d^SEM, standard error of the mean

^e^D, diet effect; M, marker effect; D × M, diet by marker interaction

^f^D, diet consisted of low (L) CP hay (CP = 79 g/kg DM); medium low (ML) CP hay (CP = 111 g/kg DM); medium high (MH) CP hay (CP = 131 g/kg DM); and high (H) CP hay (CP = 164 g/kg DM)

Also, diet affected (*P* < 0.01) the estimates of DMD determined from a combination of the 4 fecal grab samplings per day or by total collection. However, the estimates of DMD using ADIA_1234_ and APL_1234_ and the DMD value obtained by total fecal collection procedure were not significantly different (*P* = 0.11, Table 2) from each other. The diet × marker interaction did not affect (*P* ≥ 0.74) estimates of DMD.

Estimates of FO and DMD (Table 3) by ADIA, and APL using samples from different fecal sampling times (1, 2, 3, 4) and their different 2-, 3-, and 4-way combinations were not different from the measured in vivo values (*P* ≥ 0.83 and *P* ≥ 0.72; respectively). Diet had an effect (*P* < 0.01) on the prediction of FO and DMD for all internal markers while the diet × time did not impact (*P* ≥ 0.82) FO and DMD prediction.

**Table 3:** Comparison of measured in vivo fecal output (FO, g/d) and dry matter (DM) digestibility (DMD, g/kg DM) with estimates determined using acid-detergent insoluble ash (ADIA), and alkaline-peroxide lignin (APL) using samples from different sampling times and their combinations^a^

		Time of sampling^a^																	*P-*value^d^		
Item	Marker	1	2	3	4	12	13	14	23	24	34	123	124	134	234	1234	TC^b^	SEM^c^	D^e^	T	D × T
FO																					
	ADIA	4036	4069	3928	4073	4039	3945	4046	3952	4057	3962	3967	4043	3975	3979	3986	4207	100.0	<0.01	0.94	0.99
	APL	4105	3903	3907	3922	3987	3992	3995	3896	3888	3895	3954	3888	3955	3887	3934	4207	125.5	<0.01	0.94	0.99
DMD																					
	ADIA	557	554	573	551	557	565	555	565	555	563	564	556	562	562	561	539	9.2	<0.01	0.83	0.99
	APL	550	576	571	574	565	554	564	575	578	575	568	578	568	576	571	539	12.7	0.003	0.72	0.99

^a^1, sampled at 06:00 h; 2, sampled at 12:00 h; 3, sampled at 18:00 h, 4, sampled at 24:00 h. The two, three, and four-digit numbers represent combinations of the different sampling times

^b^TC, total collection

^c^SEM, standard error of the means

^d^D, diet; T, time effect; D × T, diet by time interaction

^e^D, diet consisted of low (L) CP hay (CP = 79 g/kg DM); medium low (ML) CP hay (CP = 111 g/kg DM); medium high (MH) CP hay (CP = 131 g/kg DM); and high (H) CP hay (CP = 164 g/kg DM)

In this study, all sampling times and their different combinations produced similar results that were not different from total fecal collection. Thus, fecal sampling time had little effect on the prediction of FO and DMD. No differences between measured and predicted values of DMD and FO using fecal grab samples and representative samples from total fecal collection have been reported in previous study [7], supporting the findings from this study. In their study, two fecal grab samples per day for 14 d provided acceptable estimates of DMD for individual cows with 95 % confidence [18].

## Conclusion

Time of sampling did not alter the ADIA and APL concentrations in fecal grab samples across sampling times. Concentrations were not different from those obtained from total collection of feces. Estimates of FO from grab samples at various times and different combinations of times were not different from measured FO regardless of which internal marker was used. Similarly, DMD estimated by in vivo, samples from total fecal collection, or samples from different sampling times, and all different combinations of sampling times were not different among these two internal markers. Therefore, multiple fecal samplings within a day may not be necessary to obtain a representative sample of fecal excretion by cow when ADIA or APL are used as internal markers.

## Abbreviations

ADIA: Acid-detergent insoluble ash
APL: Alkaline-peroxide lignin
ADL: Acid-detergent lignin
FO: Fecal output
DMD: Dry matter digestibility
CP: Crude protein
ADF: Acid-detergent fiber
L: Low crude protein concentration
ML: Medium low crude protein concentration
MH: Medium high crude protein concentration
H: High protein concentration
DMI: Dry matter intake
ADF: Acid-detergent fiber
NDF: Neutral-detergent fiber
